# The relationship between psychosocial factors and cognitive test performance in a South African cohort

**DOI:** 10.1002/alz.088794

**Published:** 2025-01-03

**Authors:** Anna Jane Dreyer, Sam Nightingale, Celine Le Roux, Kevin G. F. Thomas, Caroline Sabin, Alan Winston, Saye Khoo, John Joska

**Affiliations:** ^1^ University of Cape Town, Cape Town South Africa; ^2^ University of Cambridge, Cambridge United Kingdom; ^3^ University College London, London United Kingdom; ^4^ Imperial College Healthcare NHS Trust, London United Kingdom; ^5^ Imperial College London, London United Kingdom; ^6^ University of Liverpool, Liverpool United Kingdom

## Abstract

**Background:**

Accurate assessment of cognitive impairment in low‐income settings may require consideration of complex psychosocial variables (PV). Failure to consider the association of PV with biological factors, such as HIV, could lead to false classification of cognitive impairment. We investigated the impact of PV on cognitive performance in people with HIV (PWH) and without in a low‐income area of Cape Town, South Africa.

**Method:**

273 (178 PWH) participants were recruited and 185 (145 PWH) followed‐up at 1‐year. We investigated the relationship between comprehensive cognitive testing (7 domains) and 12 PV: 5 current (income, employment, assets, accommodation, mood) and 7 recalled at childhood (assets, quality of education, exposure to trauma and violence, parental education and employment), as well as standard variables typically measured in cognition studies (age, sex, years of education). Univariable and multivariable linear regression models investigated relationships between PV (clustered using principal component analysis) and global cognitive performance (measured by global T‐score). Propensity score modelling adjusted for variables significantly associated with HIV status, to determine the direct effect of HIV status on global T‐score.

**Result:**

PWH had significantly lower scores than people without HIV in 9/12 PV (ps<.015). At baseline, 8/12 PV significantly predicted global T‐score (ps<.007), which clustered into 3 components reflecting current PV, childhood PV and experience of childhood trauma. In addition to standard variables, residing in a shack remained in multivariable analysis at baseline (β = 2.40, p = .007), and lower childhood PV at follow‐up (β = 0.82, p = .035). Unadjusted, HIV status was associated with a reduction in global T‐score of 3.72 units at baseline and 2.46 at follow‐up. At baseline, after adjustment for standard variables, the HIV association was reduced by 25.8% to 2.76, and additional adjustment for PV reduced by 41.4% to 2.18. At follow‐up, adjustment for standard measures reduced by 41.9% to 1.43 and addition of PV by 56.1% to 1.08.

**Conclusion:**

PWH in this setting have lower psychosocial indices, both now and in childhood, which are associated with lower cognitive test performance as an adult. This is incompletely mitigated by adjustments for standard variables which could result in misclassification of PWH as cognitively impaired if PV are not taken into account.

